# Adapting a mobile app to support patients with anorexia nervosa following post-acute care: perspectives from eating disorder treatment center stakeholders

**DOI:** 10.3389/fdgth.2023.1099718

**Published:** 2023-05-19

**Authors:** Anneliese Haas, Agatha A. Laboe, Claire G. McGinnis, Marie-Laure Firebaugh, Jillian Shah, Anna M. Bardone-Cone, Kathleen M. Pike, C. Barr Taylor, Denise E. Wilfley, Ellen E. Fitzsimmons-Craft

**Affiliations:** ^1^ Department of Psychiatry, Washington University School of Medicine, St. Louis, MO, United States; ^2^ Department of Psychology, University of North Carolina at Chapel Hill, Chapel Hill, NC, United States; ^3^ Department of Psychiatry, Columbia University, New York, NY, United States; ^4^ Department of Psychiatry and Behavioral Sciences, Stanford University School of Medicine, Stanford, CA, United States; ^5^ Center for m2Health, Palo Alto University, Palo Alto, CA, United States

**Keywords:** anorexia nervosa, eating disorders, mobile intervention, social networking, post-acute treatment, stakeholder engagement

## Abstract

**Introduction:**

Anorexia nervosa (AN) is a harmful, life-threatening illness. Patients with severe AN often receive acute treatment but, upon discharge, experience high relapse rates. Evidence-based, outpatient treatment following acute care is critical to preventing relapse; however, numerous barriers (e.g., location, financial limitations, low availability of providers) preclude individuals from accessing treatment. mHealth technologies may help to address these barriers, but research on such digital approaches for those with AN is limited. Further, such technologies should be developed with all relevant stakeholder input considered from the outset. As such, the present study aimed to garner feedback from eating disorder (ED) treatment center providers on (1) the process of discharging patients to outpatient services, (2) their experiences with technology as a treatment tool, and (3) how future mHealth technologies may be harnessed to offer the most benefit to patients in the post-acute period.

**Methods:**

Participants (*N *= 11, from 7 ED treatment centers across the United States) were interviewed. To analyze the data for this study, each interview was manually transcribed and analyzed using components of Braun and Clarke's six-phase thematic analysis framework (Braun & Clarke, 2006).

**Results:**

Participants indicated proactively securing outpatient care for their patients, but mentioned several barriers their patients face in accessing evidence-based ED treatment. All participants had some experience using various technologies for treatment (e.g., teletherapy, self-monitoring apps), and mentioned a high level of interest in the development of a new app to be used by patients recently discharged from acute treatment for AN. Participants also offered suggestions of effective and relevant content for a potential app and adjunctive social networking component for post-acute care of AN.

**Discussion:**

Overall, participants expressed positive attitudes toward the integration of an app into the care flow, suggesting the high potential benefit of harnessing technology to support individuals recovering from AN.

## 1. Introduction

Anorexia nervosa (AN) is a life-threatening disorder that is characterized by an intense fear of gaining weight, distorted body image, low body weight, and a high drive for thinness (1). Accompanied by serious physical, social, and psychological consequences, including cardiovascular complications, infertility, osteoporosis, feelings of isolation, and anxiety (2), AN has the second highest mortality rate of any mental disorder (3). In this way, effective treatment of AN is critical.

Many patients with severe AN are often first treated in the acute setting–inpatient, residential, partial hospitalization, and intensive outpatient programs (4)–and then discharged to outpatient care. Relapse after acute treatment, however, is common; current research reports relapse rates between 31%–52%, with the highest rates of relapse in the 2 months following discharge from acute treatment (5–7). The main goals of acute treatment, and often the standard for discharge, are weight restoration and medical stabilization (8). Although these components of recovery are important, research has found that weight-based recovery is not sufficient, and full recovery involves resolution of the physical, behavioral, *and* psychological symptoms of the disorder (9). With this holistic definition of recovery, a treatment course of 3–6 months is recommended (9, 10); however, the average length of stay for patients in the US is 16 to 34 days in inpatient treatment and 52 days for residential treatment (11–13). Thus, achievement of full recovery in the acute setting is challenging and often not possible given the condensed time frame. In order to prevent relapse and achieve full recovery, high quality outpatient care is imperative.

Indeed, evidence-based outpatient treatment offers substantial support to patients with AN. For example, several studies report that adult patients show considerable weight gain, a lower likelihood of relapse, and a decrease in the overall severity of their illness during cognitive behavioral therapy (CBT) (14–17), highlighting the benefits of outpatient care. Although evidence-based outpatient therapy can be effective, the vast majority of patients with AN do not have access to outpatient providers who specialize in evidence-based ED treatment. In a study conducted by Kästner et al. (18), two of the most common obstacles preventing patients from receiving outpatient treatment were long waiting lists and low availability. Even when individuals do receive care from ED specialists, most do not offer the evidence-based interventions demonstrated to help patients achieve full recovery (19); indeed, only 6%–35% of ED providers use evidence-based approaches (19). Lack of access to treatment providers who offer evidence-based therapy perpetuates a treatment gap. Beyond this gap in evidence-based treatment access, many patients who do receive care can only attend weekly appointments (21), although many require and/or desire additional support. To fully address the needs of many individuals with AN following intensive treatment, additional avenues for treatment are necessary, such as mobile mental health interventions (22).

Although research on the efficacy of mHealth technologies for AN treatment is limited, there is considerable evidence for the benefit of mHealth technologies in the treatment of other eating disorders. For example, Fitzsimmons-Craft et al. (23) developed a digital, CBT-based, guided self-help intervention, Student Bodies-Eating Disorders (SB-ED), for those with binge-purge type eating disorders. Compared to the control group, who was given a referral to their college counseling center, those with access to SB-ED experienced a significant reduction in eating disorder psychopathology, lower rates of binge eating, lower rates of compensatory behavior use, and decreased eating disorder-associated clinical impairment, and also had higher rates of utilizing care (23). Furthermore, meta-analyses on the use of E-therapy for the treatment of eating disorders indicate a reduction in ED psychopathology for some app-based treatments (22, 24).

With regard to AN specifically, two studies evaluated the efficacy and acceptability of digital interventions following discharge from acute care. Neumayr et al. (25) tested the effectiveness of Recovery Record, an app that offers tools for self-monitoring of meals and typical ED thoughts, feelings, and behaviors, alongside feedback from a therapist, and found no statistically significant changes in ED psychopathology with use of the app, but high acceptability of the app, suggesting that patients with AN are open to utilizing mhealth technologies to further their recovery. In another study, Fichter et al. (26) assessed a digital relapse prevention program for patients with AN based on the principles of CBT and found that those assigned to the intervention group experienced statistically significant increases in weight, reduction of bulimic symptoms, and improvement of menstrual function compared to those who received usual treatment. Taken together, the results from these studies suggest the modest success of digital interventions as an adjunct to traditional treatment, highlighting a need to further explore the efficacy of coached, digital interventions for the post-acute care of AN.

Furthermore, social support has been found to be critical to recovery from AN (27, 28). Patients feel more hopeful about recovery when they develop stronger emotional connections with individuals supportive of recovery, and motivation for recovery is especially fueled by supportive relationships (34). However, many individuals working toward recovery from AN report feeling misunderstood by others, including health professionals, friends, and family (27), and as such, peer support is likely to improve recovery (28). Importantly, one scalable way in which individuals with AN could access social support from peers or others with similar experiences is *via* social media (29). Incorporating a positive, recovery-focused online community into AN treatment might be particularly beneficial as an alternative to the many pro-ED online communities that often encourage disordered behaviors (30). Although pro-ED sites contain many harmful aspects, users perceive social support as one of the key functions (31), and desire for support, interaction with others, and connecting with others with an ED are reasons individuals report for engaging with them (32, 33). These findings point to the importance of social support, particularly from peers, in the process of recovery from AN, including the need for a positive social networking outlet, where one could connect with others in recovery.

Although past studies illustrate the potential for a guided, app-based intervention for the post-acute care of AN, there are limitations to existing research. Currently, no guided, app-based interventions following acute treatment have been tested or developed in the United States; both studies on digital interventions for patients with AN were done in Germany (29). Additionally, the most promising intervention developed by Fichter et al. (26) was solely web-based. Newer, more interactive mobile platforms may provide a better user experience. Furthermore, past apps have not included adjunct social networking options, which may promote social connection and ultimate recovery in those with AN. Finally, past apps have been developed by researchers. By incorporating feedback from stakeholders—in the case of this study, treatment center representatives—the app is more likely to be useful and implemented into the treatment framework (30, 31).

Therefore, the aims of the current study are to (1) examine the discharge process from acute care to outpatient treatment, (2) evaluate how providers currently utilize technology and mental health apps for treatment of patients, and (3) gather feedback on how best to incorporate an app into the post-acute care of AN. Provider feedback will maximize the likelihood that the resultant digital intervention meets the needs of both providers and patients, maximizing potential to increase access to evidence-based care for patients with AN following discharge from acute care.

## 2. Materials and methods

### 2.1. Participants

Participants were ED treatment providers or administrators 18 years of age or older. 19 providers from 12 treatment centers around the US (all academic medical centers or private, multi-level treatment centers) that had agreed to partner with the research team on a project developing and testing an app for individuals with AN (32); trial registered at NCT05499676) were emailed a Qualtrics survey to determine their eligibility. Their emails were obtained upon their expression of interest in participating in such interviews. Of the 19 treatment center representatives contacted, 11 from 7 treatment centers consented to participating in the study. Demographic breakdown and professional classification of the 11 participants can be found in Supplementary Material Table S1.

### 2.2. Procedures

Treatment center representatives were contacted by email and asked to complete a brief online survey. Upon completion of the initial survey, eligible participants were contacted *via* email to schedule a video conference interview. Interviews were approximately 45 min in length and were conducted and recorded through Zoom. Following the study interview, each participant was emailed a $25 Amazon gift code.

### 2.3. Measures

#### 2.3.1. Demographics

Participants reported their age, gender identity, educational attainment, race, and ethnicity.

#### 2.3.2. Interest in an app-based intervention

To gauge interest in an app-based intervention, participants were asked “What is your current interest level in incorporating app-based interventions to support your patients with anorexia nervosa after they are discharged from higher levels of care?” They responded from 1 to 5, with higher scores indicating greater interest in use of an app-based intervention.

#### 2.3.3. Comfort with an app-based intervention

To gauge comfort with an app-based intervention, participants were asked “What is your comfort level regarding the integration of app-based interventions into post-acute treatment for anorexia nervosa?” They responded from 1 to 5, with higher scores indicating greater comfort with use of an app-based intervention.

#### 2.3.4. Current discharge processes and potential integration of app-based treatment

To address the aims of this study, the research team created a semi-structured interview guide that assessed each treatment center's approach to the discharge of patients to outpatient therapy, the representatives’ opinions on and experiences with technology for eating disorder treatment, and suggestions for the successful integration of technology into the treatment flow (see Supplementary Appendix A for the interview guide). To gather the most relevant feedback, participants reviewed a content outline of the proposed guided self-help app. Additionally, participants were shown screenshots of the existing ED app developed and tested by our team to illustrate the usability, style, and organization of the previous guided self-help app and solicit feedback (23, 33).

### 2.4. Data analysis

To analyze the data for this study, each interview was manually transcribed and analyzed using components of Braun and Clarke's six-phase thematic analysis framework (40). Thematic analysis is a method of qualitative analysis that works to recognize, analyze, categorize, and report patterns within data (40). The first step of this framework is to gain familiarity with the data. For this step, each interview was manually transcribed and reviewed to obtain the most accurate data. Additionally, a brief list of notes and key takeaways was compiled. For the second step, generating initial codes, each transcript was coded. In Braun and Clarke's thematic analysis, codes are aspects of data that are particularly interesting to the analyst. In the third step, searching for themes, the codes were analyzed to generate a list of themes and subthemes. Step four was the process of reviewing themes. In this step, the list of themes and subthemes was refined to better answer the research questions; the themes, subthemes, and associated data were reread to assess relevance, coherence, and consistency of the data. In the fifth step, defining and naming themes, the themes and subthemes were further refined, labeled, and compiled. For the sixth and final step, producing the report, all themes, subthemes, and coded quotes were compiled into a single document.

## 3. Results

All eleven participants completed the brief eligibility survey and expressed their levels of interest in, and comfort using, an app-based intervention for the post-acute care of AN. On a scale of 1–5, 1 being very disinterested and 5 being very interested, the average interest level was 4.27 (SD = .65). On a scale of 1–5, 1 being very uncomfortable to 5 being very comfortable, the average comfort level among participants was 3.82 (SD = .87).

### 3.1. Themes

Five major themes were identified from provider feedback: (1) participant attitudes toward and approaches to outpatient care (e.g., discharge treatment plans, referrals), (2) barriers to outpatient care, (3) participants' past experiences utilizing technology as a treatment tool, (4) participants' comfort, interest, and skepticism with app-based treatments, and (5) participant suggestions for implementing a self-help, app-based treatment for the post-acute care of AN.

#### 3.1.1. Approaches to post-acute care for anorexia Nervosa

The first theme, provider approaches and attitudes toward post-acute care, encompasses referrals to outpatient care, support from treatment centers following discharge, and nutrition and weight recommendations.

The majority of participants (*n* = 7) expressed taking proactive steps to connect patients to outpatient care, with the goal of furthering the physical, psychological, and behavioral progress made in intensive treatment. Providers noted that their treatment centers identify outpatient therapists, and when possible, outpatient dietitians, for patients prior to discharge. Participant 5 explained, “We have a discharge planner whose whole job is to…help make sure that people are going to continue their care in a substantial way after they leave.” They connect patients directly to outpatient providers and ensure initial appointments are scheduled upon patient discharge.

Although most participants reported this proactive approach of linking patients directly to outpatient providers, some participants (*n* = 2) expressed that their treatment centers take a more hands-off approach, requiring patients to initiate this process. For example, participant 4 noted “A bulk of that is…on the client end. We provide referrals but it”s…on the client to then reach out to those people to see if they have availability.” In this way, some treatment centers place more responsibility on patients to find outpatient care.

In addition to connecting patients with outpatient providers, some participants (*n* = 8) reported that their centers offer bridge programming or support groups. Bridge programming allows patients to continue seeing their treatment center therapist while they wait for an outpatient therapist to become available. Support groups, including alumni groups for those discharged from intensive treatment, also help patients navigate the transition from intensive treatment to outpatient care, and allow individuals pursuing ED recovery to connect with one another.

Along with fostering continuity of care through connecting patients with outpatient providers and support groups, treatment centers also offer weight and dietary recommendations to maintain gains made during treatment. Weight requirements at discharge vary across treatment centers; for example, participant 6, a representative from a medical stabilization unit, noted patients remain in unit if they are below 75% of their estimated body weight, but once they meet that target, they are discharged under the expectation they will continue to gain weight in future treatment. Other treatment centers set a goal weight for discharge based on premorbid weight, growth charts, BMI, and typical weight. Many treatment centers also encourage weight gain and maintenance through meal plan recommendations following discharge. Generally, patients follow set meal plans in treatment, and are encouraged to adhere to a meal plan upon discharging. For example, participant 7 indicated their patients are given a specific meal plan at discharge, but are also notified that their outpatient team may make changes throughout their care. Quotes and subthemes are described further in Supplementary Material Table S2.

#### 3.1.2. Barriers to outpatient care and further treatment

The second theme of this study encompasses participants' opinions on barriers to finding and accessing outpatient care. Several barriers to outpatient care were noted by participants, the most common of which was availability of outpatient practitioners. Participants (*n* = 9) mentioned a lack of specialized eating disorder professionals and explained that waiting lists for outpatient therapists are extensive and “The longer someone is on a waitlist the less likely they are to follow up with patient care” (P3). In many cases, it can be months before patients are able to see a therapist. One participant even expressed that, to their knowledge, “A lot of outpatient therapists are full… not accepting new patients, and they don't even have…waitlists” (P5).

In addition to availability, participants (*n* = 7) identified location as a barrier to outpatient care. Patients from more rural areas often have difficulty accessing residential and inpatient treatment, much less nearby outpatient therapists that specialize in EDs. Thus, patients are offered the next best option, including referral to therapists with other specialties.

So, location is huge. Especially in our program, what I see transitioning out, if we have someone who is a few hours away, it's harder for us to set them up with appropriate providers. It's one of those things that, if you're in such a rural area, it's sometimes hard getting those services you need, and it's sometimes at the point–it's really sad–but we have to…switch things up. For example, if someone has issues with mood… then we refer them to mood because that's all we can do. (P11)

Participants also mentioned the prevalence of financial barriers (*n* = 9), noting that available ED professionals often do not take insurance, and “The out-of-pocket cost is pretty high and there are less resources. So, if people are really relying on their insurance…they”ll be given a very long list of providers, most of whom don”t specialize in EDs, and if they do, they have a long waiting list” (P1). Another financial barrier identified includes a lack of stable income as a result of the debilitating nature of EDs, which precludes individuals from “[having] things like appropriate food to work with their meal plan upon discharge” (P8).

Additionally, participants (*n* = 6) cited patient initiative as a large barrier to following up with an outpatient team. Lack of motivation, denial, disinterest, and unwillingness to take more time off from work or school were all reasons patients did not follow up with an outpatient therapist. Even if their treatment team is driven to set up appointments for an individual, they “Get to the point where [they”re] calling and trying to set appointments up for [the patient], but [the patient] just doesn”t follow through” (P9). Other barriers mentioned less frequently include language barriers for those whose first language was not English, clinician burnout, transportation, and lack of privacy in the era of telehealth. Quotes and subthemes are described further in Supplementary Material Table S3.

#### 3.1.3. Past experiences using technology as a treatment tool

The third theme in this study is participants' past experiences and attitudes toward the use of technology during and following treatment. All participants mentioned experience with telehealth and most (*n* = 10) had experience with self-monitoring apps for ED symptoms (e.g., Recovery Record, Rise Up and Recover). When asked about the strengths of telehealth, participants cited the convenience of telehealth platforms. Most participants noted a rapid switch to telehealth during the pandemic that resulted in increased accessibility of treatment for patients:

I think it makes it more accessible…so they don't have to…drive in for a…therapy appointment. Now, they can join from work…So, I think it's been a really helpful piece in making treatment more accessible. And…especially in our IOP where we are really seeing people that otherwise wouldn't have been covered or don't live in drivable distance from the center; they are now able to receive support that they wouldn't have otherwise. (P2)

Participants also highlighted greater accessibility of treatment center-implemented support groups and bridge programming, that patients could engage in *via* telehealth upon discharge.

Along with greater accessibility, participants explained that patients, with telehealth, were able to experience “more authentic therapy” (P2), as they did not need to wear masks with teletherapy. Furthermore, as patients were typically in their home environments, participants expressed that patients tend to feel more comfortable with food exposures (P6). Participants also noted a preference for telehealth themselves, noting the ability to work from home as a benefit.

In addition to sharing the benefits of telehealth, participants shared drawbacks. A commonly reported drawback was the unreliability of technology; many participants noted experiences of glitches and connection problems while using a telehealth platform. Furthermore, several participants mentioned that patients could be less engaged while using a telehealth platform as opposed to treatment in person. One participant explained that:

Telehealth can lead to this…minimization of the importance or… separateness of the therapy session from the rest of life. That's not always necessary, but I think in some cases especially, if the patients are really struggling or having a hard time getting on board with treatment, it can feel a little bit more difficult to get that engagement over the screen…or make it feel as valued. (P6)

Participants also expressed that telehealth makes it easier for patients to hide food, lie about weight, and engage in disordered eating behaviors. Finally, with telehealth, participants reported that some patients find it “harder to be vulnerable and open” (P1).

Participants shared strengths and weaknesses of meal tracking/self-monitoring apps (e.g., Recovery Record, Rise Up and Recover), expressing a high regard for the practicality of the apps, the ability to export PDFs from the apps, and additional coping skills offered in apps. As described by one participant, “It”s something that's more subtle, it's so easy for them to…be at school on their phone logging their meal; no one knows what they're doing, right, vs. this big old notebook with their last 20 meals, right?” (P9) This ease of use was a benefit of apps consistently noted by participants.

Few weaknesses of apps were mentioned. The main limitation of apps, as cited by participants, is the interface layout. Specifically, several participants expressed available digital resources for EDs tend to be clunky and unsophisticated. Further, one participant expressed that certain apps are no longer being updated and “If you try to suggest anything you get a sorry email “we are no longer working on this app”” (P9). A final weakness of apps mentioned by one participant is cost. Although most apps are free, some cost money, adding a financial barrier to care. However, the participant who expressed the financial burden these apps might impose still expressed that apps are preferable to pen and paper (P7). Quotes and subthemes are described further in Supplementary Material Table S4.

#### 3.1.4. Comfort, interest, and skepticism in App-based treatments

The fourth theme in this study consisted of participants' overall interest in and skepticism of app-based interventions. All participants expressed a high level of interest in app-based treatments to support patients with AN following discharge with acute care, noting numerous benefits. One participant explained that “any kind of evidence-based…eating disorder clinician-reviewed resources that we can provide that are easy to access could pose such a benefit to our patients, especially given…the increased need and lack of resources” (P2). Another participant also expressed excitement about this technology's potential:

It's one of the things…we can't escape, and we shouldn't. I have this philosophy that…we shouldn't escape it, we should embrace it. So…if there's a tool out there that can be super helpful and can provide…accountability for our clients, support for our clients, help us or help our clients get more success in our program, I think that's what matters (P11).

Further enthusiasm stemmed from a recognized need of continued evidence-based treatment for patients receiving outpatient care. One participant explained that restoring weight and stabilizing patients is a strength of acute care; however, “finding ways to help people be successful in maintaining that for long enough for it to, through whatever mechanism, to get to be easier to maintain,” is the next goal (P10), and app-based treatments hold much potential to continue to support patients in that journey.

Regarding comfort with use of apps, participants shared that comfort in an app is heavily dependent upon level of familiarity with that app. Participants stressed the importance of testing an app themselves before introducing it to their clients. Several participants also expressed that change is hard, and sometimes “At the end of the day, you just don't have the extra time that goes with learning one extra tool” (P7), which is a barrier to ultimate comfort in and adoption of an app-based treatment.

Other participants also expressed skepticism regarding app-based treatments, particularly surrounding their ability to be successfully implemented. For example, one participant noted:

There's been a fair bit of stuff that has been presented to be simple, painless and/or free… but for the most [part]…at least one and often more than of those points has failed to be met (P10).

Finally, one participant explained that the relationship between a therapist and their patient could never truly be replaced by an app, and at most, apps can supplement treatment but not serve as treatments themselves (P5). Quotes and subthemes are described further in Supplementary Material Table S5.

#### 3.1.5. Strengths and suggestions for improving the implementation and integration of a guided self-help, App-based treatment and adjunct social networking component for the post-acute care of An

The fifth and final theme of this study is participant feedback on implementation, development, and integration of a guided self-help app, based on SB-ED, into the AN treatment framework. Participants, when shown screenshot of the proposed guided self-help app for AN, described several strengths, including the ability to both message and video chat a coach, as well as the usability of the app, describing it as “user-friendly” (P3), “clean” (P2), “streamlined” (P11), and “easy to navigate” (P2). Another identified strength was the comprehensive nature of the app; participants were impressed by the mix of psychoeducation and interactive activities throughout the app. One participant mentioned that the app contains the content that he covers in bridge sessions: “It is literally what I do in the bridge sessions. It's actually… I'm finding it…almost humorous… it's awesome that a robot could potentially replace me, which would be great for my caseload” (P9). In this way, participants noted multiple strengths that indicate high usability and acceptability of a guided self-help app for the post-acute care of AN.

Participants also offered feedback on the proposed adjunctive social networking component, and overall, were optimistic about including this alongside the app to further social support. One of the participants noted, “[An eating disorder]…loves secrecy…so it can feel isolating, and like you”re the only one dealing with this, because by nature, you”re trying to hide what”s going on. I think having that network of individuals to be like “Hey there”s other people dealing with this,” can be really helpful for clients to not feel as alone.” (P4) Another participant noted:

I support it fully. I think there is very much a fear about putting a bunch of folks with anorexia in the same room; I'm not in that camp…I think the people that want to get better will use that appropriately, the people that don't won't. As long as it's being monitored and someone is keeping tabs on what's being posted and how people are interacting, I see that being really beneficial” (P9).

Thus, participants saw much potential for the social networking component to be helpful, so long as it is strictly monitored. These strengths are described further in Supplementary Material Table S6.

In terms of suggestions for an app-based treatment for the post-acute treatment of AN, the most common recommendation was to make the app engaging and interactive. Participants suggested incorporation of games, interactive learning activities, multiple forms of media (podcasts, videos, etc.), and a progress bar or reward system to support app users' engagement. Multiple participants also emphasized the importance of integrating different therapeutic approaches (e.g., CBT, Acceptance and Commitment Therapy) so that patients might focus on what resonates with them.

Participants also offered recommendations for specific sessions or activities, including a session on triggers to prepare patients for the transition “To the real world where…people talk about stuff like the gym or calories all the time” (P4), and sessions for body image, body exposures, and food exposures. Furthermore, participants encouraged inclusion of sessions for patients to reflect on reasons for recovery and continued use of the app.

Regarding the practicality of the app, participants noted that the ability to upload information from therapy sessions and integrate assessment data from the app and therapy sessions would be beneficial. Participants also expressed a desire for an app that is “Constantly being updated” (P9) and “That takes feedback from both clinicians and the individuals using it” (P9), so that users can access new content, and app experience can consistently be improved. Participants also suggested “clear-as-day” tech support (P9), as well as a comprehensive training for app use. Overall, participants desired a well-functioning app that would be straightforward to use. These suggestions are described further in the Supplementary Material Table S7.

In terms of when to introduce the app into the treatment flow, participants had opposing views. Many noted that although the app's psychoeducation components could supplement higher levels of care, the coaching and self-monitoring would not be appropriate at that point in time. Thus, participants were ambivalent about integrating the app into higher levels of care, with most ultimately deciding that the app would be most beneficial in the outpatient settings.

Participants were also mixed about who should coach the app. Some were in favor of utilizing in-house providers because the therapist and app user “already had that established relationship” (P6); however, participants noted that this would not be realistically feasible given liability issues, clinician burnout, billing issues/reimbursement, and time constraints. Thus, many participants noted the benefits of having third-party providers be coaches:

I think it would be more realistic, less burdensome for the clinics and centers to have a third-party outsider. I recognize that there's a potential for reported drop there, and, at the same time, I think about, sometimes what happens for some of our folks is those bridge sessions become, I don't want to say like kind of an excuse not to follow through with outpatient, right, but like if it becomes too comfortable, too personal, then it does become… “Oh well this is just going to be my therapy,” and it's like “no actually, more is still needed,” right? Like the work has just started. So, I think that…having it be a third-party person that they could build some rapport with potentially, over the course of the time that they're using this app, but they wouldn't get to the point where it's comfortable like they may just try to stay there. I think that could be really helpful (P9).

Overall, participants expressed numerous strengths and suggestions for the development, implementation, and integration of a guided self-help, app-based treatment to support patients with AN following intensive treatment.

## 4. Discussion

The present study used semi-structured interviews to collect experiences, insight, and suggestions from treatment center representatives to inform development of an app for the post-acute care of AN. Treatment center representatives were recruited from academic medical centers and private, multi-level treatment centers that are the current options for intensive ED treatment in the United States. Findings from this study offer an account of participants' current attitudes and approaches to post-acute care, their opinions on barriers to treatment, their past experiences with technology in treatment, their attitudes towards app-based treatments, and their suggestions for the mobile health app our research team is currently developing. The feedback from treatment center representatives supplements feedback received from individuals from the proposed patient population (i.e., individuals being discharged from acute treatment for AN) who would be using the app (Laboe et al., in preparation). The use of participatory research, through which feedback from individuals affected by the research was garnered, will facilitate successful implementation of the app into the care flow (30, 31).

The first goal of the study was to more fully understand, and report on, the transition from acute care to outpatient care. Aligned with past literature (8), several participants explained that acute care aims to get patients medically stable and back to a healthy weight range. Weight restoration, although an extremely important part of treatment, does not reflect full recovery; full recovery is also based on a reduction of ED behaviors and a decrease in intrusive eating disorder thoughts and feelings (22). Some participants explained that even weight-restored patients may stay in acute treatment for longer if further care is required, but this was not the norm. The majority of participants explained that their patients are discharged from treatment centers once they hit their goal weight range, especially in hospitalization programs. The psychological components are addressed in acute treatment, but participants expressed that outpatient care is critical for sustained progress. Thus, most participants and their treatment centers take a proactive approach to outpatient therapy, with many acute care providers setting up their patients' outpatient team for them as well as consistently communicating with the team to ensure individualized and quality care for their patients. Many treatment centers also provide online support groups or bridge programming to their patients to offer support as they wait for access to outpatient treatment.

Although most patients with AN receive the recommendation of continuing outpatient care, as reflected in the responses from these participants, a good portion of patients do not have access to high-quality outpatient providers due to extremely low availability (18). Participants in the current study expressed that, even if patients find access to a provider, the likelihood of that individual being trained in evidence-based care is low, further confirming past literature (19). Other commonly cited barriers included location, insurance and financial barriers, and the ambivalent nature of AN, all of which further compound difficulties in accessing quality treatment following acute care. Participants could not offer specific percentages on how many of their patients followed up with outpatient care, but each noted it was not 100%; oftentimes, patients that did not engage with outpatient therapy returned to higher levels of care, which aligns with previous literature on the poor outcomes and high relapse rates associated with AN (41, 42).

Though the barriers to treatment noted by participants of this study are consistent with past literature (18), technology-based treatment tools may have the power to address these gaps in a real and effective way. This potential capability sparked the second goal of this study, which was to examine how providers currently utilize technology and mental health apps in their treatment framework. Due to the COVID-19 pandemic, a rapid shift to telehealth occurred in almost all sectors of the professional world and this shift took place within ED treatment as well (43). All participants in the current study had experience with telehealth, and most had experience with tracking apps. Though participants only introduced telehealth services because of the COVID-19 pandemic, many continue to use, and have a high regard for, teletherapy today. The most commonly noted strength of telehealth was the convenience and accessibility that it offered; because evidence-based ED treatment is so difficult to access, teletherapy has made treatment possible for more of their patients in rural areas. However, participants commented on a series of telehealth weaknesses as well. For example, many participants saw their patients waver in accountability once teletherapy became the dominant form of therapy. Additionally, therapy still costs money and clinicians still have waitlists, even when their work has been moved to a virtual setting.

Thus, telehealth may address some barriers, but other technologies must be utilized to close the gap between acute and outpatient care in a sustainable and effective way. For example, app-based interventions allow users to easily access services when it is convenient for them and at a low cost (44). The self-guided nature of apps helps to lighten the load for clinicians. The participants who had experience with this type of self-help app or guided self-help app considered the comprehensive nature of the apps as the greatest strength. For guided self-help particularly, where individuals using the app also have the support of a coach, participants acknowledged the potential for reliance on a coach to preclude individuals from seeking additional support from an outpatient provider. Thus, having coaches encourage users to seek professional help is critical. Participants also noted the importance of making the coaching feature easy to access, user-friendly, and appealing. Furthermore, they saw the availability of a coach as an opportunity to promote social support, which has been found to be essential during recovery from AN (27, 34, 45, 46).

The final goal of this study was to gather feedback on how to best develop an app-based intervention and adjunctive social networking component for the post-acute treatment of AN, and then integrate it into the post-acute treatment framework for AN. The main suggestions mentioned in the interviews fell under one of three categories: suggestions for improving engagement, suggestions for specific content, and general hopes for how the app would look, function, and offer technological support to users. Current research emphasizes that engagement often centers around simplicity, validity, and customizability (47, 48). These principles were reaffirmed in this study; however, participants also recommended more specific suggestions like the inclusion of games, voice-guided body and food exposures, videos, podcasts, interactive tools, and the ability to set notifications for the app to increase engagement. Additionally, participants suggested that sessions incorporate multiple treatment modalities. These sessions should allow users to learn about and reflect on real-world triggers, body image, and reasons for recovery. Lastly, the technological burden should be low and technological support should be clear, concise, and effective.

Participants additionally provided feedback on the adjunctive social networking component, emphasizing that a heavily moderated social media group may be beneficial. Specifically, participants emphasized the isolating nature of EDs, and how support through a social media group might help individuals feel less alone. Indeed, individuals with EDs seek connection with other individuals with EDs (Csipke & Horne, 2007; Wilson, Peebles, Hardy, & Litt, 2006), and social media offers a scalable way for individuals with EDs to access such social connection (Aardoom, Dingemans, Boogaard, & Van Furth, 2014). Participants also suggested that any social media group be strictly monitored given the tendency for individuals with EDs to compare with one another. This is also important given the prevalence of pro-ED online communities that may encourage disordered eating behaviors (Rouleau & von Ranson, 2011). Suggestions for the app and social networking component will be incorporated into our own app-based intervention for the post-acute care of AN with the hope that provider feedback will enhance the efficacy of the app and offer more benefits to the user.

Though the present study offers many important findings, there are still several limitations. Of providers interviewed, all were white and not Hispanic or Latino. Additionally, of the eleven providers, eight were female. The lack of diversity in this sample does not allow us to generalize results to all acute-care providers. In the future, researchers should work to increase the diversity of the sample to obtain the most representative information. Another limitation is the way the future app was explained to participants. The brief overview of the app and the screenshots from the past SB-ED app, which the current app is being adapted from, do not offer as much information to the participant as an app prototype would. Future research should keep this in mind and try to incorporate screenshots from the specific app for which participants are providing feedback, or even offer providers the chance to review prototype apps. Finally, the thematic analysis is limited by the fact that it was conducted by one rater.

The findings of the present study offer a myriad of implications for the future of app-based treatment. The insight, feedback, and suggestions for improvement will not only refine and enhance our own app, but can also be used to help app developers and other research labs to design future apps for the treatment of eating disorders, and for mental health treatment in general. Demand for treatment is high and supply is low for most psychological disorders in the world today (49, 50). Creating app-based interventions to supplement high-demand in-person treatment could change the way mental healthcare is deployed. However, the key to developing quality app-based interventions is engaging individuals who will be most implicated by the intervention: app users (Laboe et al., in preparation) and treatment center representatives. By gaining insight and feedback from treatment center representatives, app developers and researchers can harness the power of digital technology and create the most effective, practical, and powerful tool to close the gap between acute treatment and outpatient follow-up for AN.

## Data Availability

The raw data supporting the conclusions of this article will be made available by the authors, without undue reservation.
