# Supplementary material for: Adapting a mobile app to support patients with anorexia nervosa following post-acute care: perspectives from eating disorder treatment center stakeholders
Source: Front Digit Health. 2023 May 19;5:1099718. doi: 10.3389/fdgth.2023.1099718 (PMC10235779; doi:10.3389/fdgth.2023.1099718)
Supplement: Supplementary file 1 [file Datasheet1.pdf]

Supplementary Material

1 Supplementary Tables

1.1 Table 1

Participant Breakdown l

Participant ID	Job Title	Highest Degree Earned	Field of Study	Professional Licensure	Treatment Center Type (7 Treatment Centers Represented)
P1	Clinical Psychologist	PhD	Clinical Psychology	Licensed Psychologist	Academic Medical Center (1)
P2	Nutrition Manager	Master's Degree	Nutrition and Exercise Science	RD	Private, Multi-Level Treatment Center (2)
P3	Supervisor for Outpatient Programs	Master's Degree (MS)	Clinical Rehabilitation and Mental Health Counseling	LPC	Private, Multi-Level Treatment Center (3)
P4	Behavioral Health Therapist	Master's Degree	Social Work	LCSW	Private, Multi-Level Treatment Center (3)
P5	Clinical Director	Master's Degree (MSW)	Social Work	LCSW	Private, Multi-Level Treatment Center (4)
P6	Associate Clinical Professor, Inpatient Psychology Services Director	PsyD	Clinical Psychology	Licensed Psychologist	Academic Medical Center (5)

P7	Clinical Fellow	PhD	Clinical Psychology	Licensed Psychological Associate	Academic Medical Center (6)
P8	Primary Therapist	Master's Degree (MS)	Mental Health Counseling	RD	Private, Multi-Level Treatment Center (4)
P9	Behavioral Health Therapist	Master's Degree (MS)	Clinical Rehabilitation and Mental Health Counseling	LPC	Private, Multi-Level Treatment Center (3)
P10	Psychiatrist	MD	Medicine	MD	Private, Multi-Level Treatment Center (7)
P11	Registered Dietitian	Bachelor's Degree	Nutrition	LPC, NCC	Private, Multi-Level Treatment Center (3)

1.2 Table 2

Theme 1: Participant Approaches to Post-Acute Care for AN

**Theme**	**Subthemes**	**Frequency of the Subtheme (# of participant comments about the given subtheme)**	**Illustrative Quotes**
Approaches to Post Acute Care for Anorexia Nervosa	Proactive approach to outpatient care	7	"From…the moment they admit to PHP, we're already… [asking] 'do you have an outpatient team established?' If so, we're already communicating from admission with them… that's something we're following up on very regularly both in our sessions and in our treatment team meetings" (P2).<br><br>"We do have a discharge planner whose whole job is to… help make sure that people are going to continue their care in a substantial way after they leave" (P5).

	Patient initiative is required to make the connection with an outpatient therapist	2	"A bulk of that is…on the client end. We provide referrals but it's… on the client to then reach out to those people to see if they have availability" (P4).
	Many treatment centers offer bridge programming or alumni/parent support groups	8	"The adult program has…a relapse prevention group…It's essentially a one-hour therapy group where people can come who discharged from our program and it's just getting support. I don't know if before COVID it was in person, but now we are doing it online" (P1).<br><br>"We've created a bunch of bridge programming for patients… one of our psychologists can meet with families for up to three 30-minute sessions post discharge, or even if they're just in the medical setting, and they haven't had any treatment yet, the medical team can refer them for consultations with their mental health team" (P6).<br><br>"Another resource we have is an alumni support group, so after the patients are discharged, for their lifetime, as long as they want it, they're allowed to attend a weekly virtual group that's also led by an ED specialist" (P8).
	Dietitians are recommended or referred on a case-by case basis	3	"Different adult patients need different levels of support with their dietitian. The adults are more likely to have more involvement with their dietitian in their treatment, especially directly, and if an adult feels like they haven't learned what they needed to learn to help…carry them or help them maintain their gains when they discharge, then we would provide referrals for an outpatient dietitian" (P1).

	Mixed modalities in terms of meal plans; generally, not prescriptive	3	"Our dietitians will provide them with…basic guidance around meal planning. They don't use exchanges; they don't use calorie counts for the most part. Primarily they just focus on strategies to increase nutritional density and give a bunch of different kinds of examples of high density snacks or other strategies to…boost nutrition" (P6).<br><br>"We're doing the exchange system here… before they're discharged, they, at least for several days, step down to their maintenance, so they kind of know what that looks like. And then they're given that plan physically, or by email, like here's the exchange system for your maintenance plan, and usually there's quite a bit of education that's provided to families or parents for how to maintain, so the dietitian works with them on helping them understand how to use the system once they're out of the hospital" (P7).
	The majority of patients follow up with outpatient therapy	7	"I don't know the statistics but…pretty much all are recommended for continued outpatient care. I do know there are some to a decent portion of clients who don't follow through, and we'll see them then again in the higher levels of care because they didn't follow through with outpatient referrals for whatever reason and then things get worse, and so they come back because they need that higher level of care again" (P4).<br><br>"If they complete treatment with us, and they don't leave us early, they are very, very likely to follow through with the plan. The one thing I can say is if we have anyone… leave early or give up or is like "I'm done, I don't need this," that's where we [say] 'ok they are probably not following up'" (P11).

	Discharge weights are based on a number of factors and depend on the treatment center in question	6	"We use goal weight ranges here so… we set a weight range and typically strive for the middle of the range as…a marker… but then if they get to that weight and they're still super entrenched, we might…come back to whether that is the best weight range for them" (P1).<br><br>"So typically, we try to restore patients to above BMI of 19. If they were underweight, then…they will typically leave with their BMI at least 19 or more. Sometimes they will reach their goal weight, but they stay in the program for a little longer for other reasons or for other symptoms, and so we might see …them reach a higher weight, but I would say 19 is minimum" (P7).<br><br>"Kind of all over the place, but I would say we tend to keep people in the hospital if their weight is below 75% of their estimated body weight. So, we look at their…historical growth records, and we calculate…where we think they should be if they were to be weight restored, recognizing that that's an estimate. But then if they're below 75% of that, then we would tend to keep them in the hospital" (P6).

1.3 Table 3

Theme 2: Barriers to Outpatient Care

Theme	Subthemes	Frequency of the Subtheme (# of participant comments about the given subtheme)	Illustrative Quotes
Barriers to Treatment	Insurance	8	"I don't know if this is specific to this region, but a lot of the outpatient therapists for EDs do not take insurance, so the out-of-pocket cost is pretty high and there are less resources. So, if people are really relying on their insurance, companies often… get the run around…They'll be given a very long list of providers, most of whom don't specialize in EDs, and if they do, they have a long waiting list. So, I definitely get the sense that there are a lot of barriers unless someone has a lot of financial resources" (P1).<br><br>"I mean right now with the increase in need, a lot of people are just on wait lists… and especially if people are wanting to use their insurance, which I don't blame them. A lot of the insurance-taking providers are on really long waitlists" (P2).<br><br>"Biggest one is insurance. Just finding a place that

			will take our patients insurance is a nightmare” (P7).
	Other financial considerations	1	“Sometimes in terms of other resources…even food if we have people who don't have a stable income because they've had an eating disorder and are unable to support themselves, they may not even have things like appropriate food to work with their meal plan upon discharge so that can also be a limitation” (P8).
	Availability of evidence-based ED providers	9	“There aren't a lot of providers, especially in the outpatient realm, who “specialize” in eating disorders” (P4)<br><br>“We don't have good access to care. And I don't know the numbers off the top of my head, but I would say the vast majority of our patients end up waiting to get into treatment” (P6).<br><br>“The first barrier is definitely the current demands. I think that there has always been a limited supply of eating disorder professionals in the communities… so, the biggest hurdle right now I think is really high demand and a really low supply” (P9).<br><br>“I think clinician burnout is a huge barrier…Through the pandemic, clinician burnout has been really high” (P2).
	Location	7	“The biggest barrier is just accessibility and especially when they get outside the main city…like if they live in the suburbs…that availability goes down significantly even more” (P4).

	A lack of patient initiative	6	“[Due to] the ambivalent nature of the disorder, patients…don't see a need, don't want to continue” (P2).<br><br>“And I think, of course, there are individual and family factors that are barriers…[including] motivation, understanding of eating disorders, acceptance of the diagnosis, and then features of the illness that make change difficult… such as fear of weight gain, and not wanting to …step away from life, which I think is also becoming more and more of a barrier as things start to open up again in this post-pandemic era. People don't want to sacrifice going to school or working or …doing other things that they…put on hold for a long time” (P6).

	Language	1	"We also have a lot of families who are not English speaking, and so finding culturally relevant and appropriate services for them in their native language is also challenging" (P6).
	Transportation	1	"The other barrier we run into for folks is just transportation, if they are able to get somewhere" (P3).
	Privacy during virtual programs	1	"I think for individuals, a big barrier that we've seen is just space and privacy if they are doing programming, even if it's outpatient, from home… it definitely makes it more challenging for folks to have that space to do therapy" (P3).

1.4 Table 4

Theme 3: Participants' Experiences Using Technology as a Treatment Tool

**Theme**	**Subthemes**	**Frequency of the Subtheme (# of participant comments about the given subtheme)**	**Illustrative Quotes**
Past Experiences Using Technology as a Treatment Tool	Strengths of telehealth	10	"People being able to access Zoom from wherever definitely increased flexibility" (P1).<br><br>"I think [being able to see someone's face on telehealth has] provided a lot of space to have more authentic therapy" (P2).<br><br>"It…reduces some of the barriers to accessing treatment" (P6).<br><br>"I also like that [my] patients can access care from pretty much anywhere, so if a patient lives in the middle of nowhere, and they don't have any therapists around, they can still see me. So, I think access to care is great" (P7).
	Weaknesses of telehealth	7	"I feel like sometimes… it was easier for there to be less engagement. If someone wasn't feeling very engaged, I feel like they could disengage easier than they would have been able to in person" (P1).<br><br>"Connection issues sometimes come up" (P1).

			"I find telehealth a lot more draining than in-person visits because I watch myself on camera all the time; I have to be mindful about how I appear on camera; I have to make sure I'm sitting straight and squared in the little camera box and I just find that more more draining on myself" (P7).
	Strengths of tracking apps	8	"I would say…what's nice about it is that you can take pictures, so that you don't have to… type up exactly what's served, visually the dietitian can just see it" (P1).<br><br>"I like that it tracks all the different symptoms, so it asks the patients have they binged, have they purged, have they used laxatives? All the different things that you can set. I also like that it actually provides patients with coping strategies, so whenever they report a behavior, it will pop up with a coping skill and I think that has been helpful for a lot of my patients" (P7).<br><br>"So those apps have all been really helpful for clients in just a number of different ways, and there are things that they can just kind of pull up whenever, however, and wherever they need it, and …go with it. So, I have a lot of clients who enjoy those apps, and participate in them, and feel like they're really helpful to use" (P8).<br><br>"Folks had a lot of really great resources at their fingertips, a lot of psychoeducation that otherwise we'd be kind of like collecting from different places and trying to get to them" (P6).
	Weaknesses of tracking apps	3	"I don't get the sense that it's…the most sophisticated app, so it might be kind of clunky or inconvenient if you are trying to input some information other than the photo. I'm not sure how…sophisticated the interface is" (P1).<br><br>"I know the developers aren't actually actively working on it anymore...If you try to suggest anything you get a sorry email "we are no longer working on this app," so it's not the best tool for that reason. We've had some folks where I think their phones have been incompatible" (P9).<br><br>"I would say that I don't remember what the data ended up looking like around that, but people probably didn't use that chat feature as much as we would've liked" (P6).

	Tracking apps are often used following discharge and throughout outpatient therapy	4	“I know that most of our outside referrals… for dietitians use [tracking apps] … So, if folks follow up with our referrals, then yes, that typically is implemented” (P3).

1.5 Table 5

Theme 4: Participants’ Comfort, Interest, and Skepticism in App-Based Treatments

Theme	Subthemes	Frequency of the Subtheme (# of participant comments about the given subtheme)	Illustrative Quotes
Comfort, Interest, and Skepticism in App Based Treatments	High level of excitement and interest in an app for the post acute treatment of AN	9	“It’s a great idea, especially for our younger population who are so into their technology and they're aware of it and they are good at using it, it would be especially useful for them because they… would find it more accessible” (P8).<br><br>“I'm always looking for ways to integrate technology, especially because… for our folks, especially with anorexia, that we see in the clinic…a part big part of that motivational factor is…having a means to check in more frequently, having an opportunity for support when you need it, on the fly” (P9).<br><br>“I feel like we need to be utilizing this technology and taking steps forward and not steps back, and I feel like by making apps …it's going to allow people, even long term… to utilize these tools and have this with them (P11).
	An increase in familiarity with an app would increase comfort in the app	6	“There's not… an app that I'm familiar with… I've never had training in it, and I've never had the experience of doing it” (P1).<br><br>“I am good with technology, but I am sure there are areas that I could be better at so I think if someone were talking about an app and how it works, I could get that” (P2).

	Skepticism in technology can prevent a high level of comfort in an app-based treatment	3	"I do a variety of types of therapy, but my training is as a psychodynamic therapist, so I guess part of my bias is a belief that the relationship is a big part of what helps in therapy… I don't know how it works, but I have some skepticism of technology" (P5).<br><br>"People try really hard to make the app work. Kind of like they're trying to please the computer… or I don't know. So that's part of what [my] skepticism is about -- can…the relationship really be replaced by technology?" (P5)

1.6 Table 6

Strengths of the Proposed Guided Self-Help App and Adjunctive Social Networking Component for the Post-Acute Care of AN

Strengths	Frequency of the Strength (# of Participants that Offered the Same Strength)	Illustrative Quotes
Simple and clean layout of the app	3	"It just looks very… clean… as far as being simple. I think it …looks like it would be easy to navigate" (P2).<br><br>"It seems like it is very user friendly. I mean even with just that quick tutorial, I can connect and figure things out, so it seems like it's very easy to use and the layout is very nice and calming as well, which is also nice too" (P3).
Feasibility of the app	4	"It seems like it's got a very…simple user face and seems…pretty easy to get around the app" (P1).<br><br>"It didn't seem confusing. So I know some apps… can be confusing and annoying and I definitely feel like you…streamlined it, so it looks nice" (P11).
Coaching component	7	"It seems nice that there's…a component where they can get support, reach out to their coach" (P1).<br><br>"I also like the idea of not only having text-based messaging between you and the coach, but also the ability to video chat if needed or if necessary, in those moments where that might be more helpful than like a text feature. I think those are… the main points. I think overall it seems like a really good app and could be really helpful for clients post that acute care" (P4).

Comprehensive and expansive in the tools available to the user	4	“I think it's clear that there's a lot of different features and different ways that people can use the app, and so I like that it can be very…self-directed, and that people can kind of take and leave what they choose, so I think that's super helpful” (P6).
Interactive nature of the app	1	“I think trying to figure out ways to really help people have the experience of practicing skills rather than just reading about them… so I like the exercises…so you can enter a thought, and then you can work on challenging it, so trying to build an algorithm that really helps people really make use of those skills” (P6).
Resource page	3	“I like how you list resources for emergency needs or you address the comorbidities and like the co-occurring issues, because as you know… eating disorders don't just form in a vacuum; there's other things that come up too” (P7).<br><br>“I like that there's resources at their fingertips; that …if they need anything in a…crisis, they know where they can turn” (P6).
Subtlety	1	“I think for some [clients], if I were to hand them an old school self-monitoring record on pen and paper, they’d be like “mmmmmm,” right? But having an app and having it be something that's more subtle, it's so easy for them to…be on their phone logging their meal; no one knows what they're doing, right? Versus this big old notebook with their last 20 meals” (P9).

Heavily monitored social media component	5	"I definitely think it can be helpful. I think especially with eating disorders; it's one of those diagnoses that loves secrecy… so it can feel isolating, and like you're the only one dealing with this, because by nature, you're trying to hide what's going on. And so I think having that network of individuals to be like "hey there's other people dealing with this," can be really helpful for clients to not feel as alone…especially when we have clients who don't necessarily fit the mold that society thinks of when they think of an eating disorder. On the other hand, I think definitely having the heavy moderation would be important because I think there's also a competitive nature sometimes…within the eating disorder world of trying to be sicker or trying to…compete with other people about behaviors" (P4).<br><br>"I support it fully. I think there is very much a fear about putting a bunch of folks with anorexia in the same room; I'm not in that camp…I think the people that want to get better will use that appropriately, the people that don't won't. As long as it's being monitored and someone is keeping tabs on what's being posted and how people are interacting, I see that being really beneficial" (P9).

1.7 Table 7

Suggestions for the Proposed Guided Self-Help App for the Post-Acute Care of AN

**Suggestions**	**Frequency of the Suggestion (# of Participants that Offered the Same Suggestion)**	**Illustrative Quotes**
Make the app engaging and interactive; incorporate games and tools to increase engagement and motivation	4	"I don't know how you would do this, but it's just a thought. With our program currently, like during meal times, we… encourage patients to…play different table games… You don't need anything else besides knowing how to play the game to play them, but…something like trivia that could be a really good game…to play, and so if the app has something like table games…that [could encourage] skill usage and things that are helpful for treatment and maintaining recovery" (P1).<br><br>"I think one thing that I would wonder is if there could be…a way to keep people engaged, like I always wonder with apps…what's gonna… keep them coming back to the app? And I think… it could be different for different people depending on the population" (P7).

Incorporate other treatment types into the app	4	Different people respond well to different messages - and so none of them are better or worse than others but having…a variety to focus on different things [could be helpful]" (P2).<br><br>"I think…it can be hard to fully tailor an app to each individual, but I think that for many people, the idea of having..everything kind of consolidated into one place…would make my job a lot easier rather than trying to…pull resources from different places and send it to them" (P6).

Incorporate sessions on real world triggers	3	"It's really difficult to go from a high level of care to…the real world, especially because within our clinic… there are a lot of..topics off the table that we don't talk about in group settings: for example, specifics of behaviors… Going from that environment, then to the real world, where…people talk about this stuff all the time – I think having that additional support can be helpful…in those moments" (P4).
Offer support or alternative resources to those that cannot (or choose not to) access technology	3	"Clients who are older…may not have the technology awareness or desire...to [engage in an app]… so what are the resources available for them? That would be kind of something to [consider]... how to make it accessible for them, even if they don't have those resources" (P8).<br><br>"I get some folks that are a bit older who are a bit less tech savvy and trying to navigate that with them is just very uncomfortable. So, I think part of it, if we're looking for solutions on how we could address that, having…some sort of a tech support available with the application would be super awesome for everyone" (P9).
Incorporate sessions on body image and self love	2	"Make the content be kind of grounded in more of a "health at every size," body acceptance or in body neutrality, anti-diet culture …philosophy. I think pulling from some of those resources and content might be helpful to…think about how to reshape the culture around it" (P6).<br><br>"Something that I often do is I tell my patients to… stop following diet accounts on social media and start following body positive accounts, so integrating something along those lines, like 'hey here's ideas for different accounts to follow; (P7).<br><br>"I actually do nutrition groups based on body image ... I think that could be beneficial, maybe to have… some tools 'cause a lot of people, at least recently in our program …they feel a lot of body image distress" (P11).

Incorporate body exposures and food exposures	1	"I think… integrating specific exercises, which you might already have…like mirror exercises or body exposures…even guided exercises…you could do, depending on again the cost of this…. Sometimes when I do… a mirror exposure with a patient, I have them stand in front of the mirror and I guide them through the exercise, but theoretically you could record that and have the app guide the patient through" (P7).<br><br>"And I think similarly with food… So maybe making a hierarchy of foods that they're afraid of, and then helping them…check off items in the hierarchy" (P7).
Use language and tools to help users normalize their relationship with food	2	"My opinion is that it's hard to have a healthy relationship with food and your body whenever there's…that transactional or…number-based method. So I feel like an app would have to have some kind of way to not focus so much on numbers" (P1).<br><br>"Another thing that my patients find really helpful is getting visuals of plates, of what a normal plate should look like… so I think an app is uniquely positioned to actually have pictures in there… Like a lot of my patients are like 'Oh I didn't know that a healthy plate would contain…this many carbs,' for example. So challenge…their idea of what they think a healthy plate looks like versus what a dietitian will say a healthy plate looks like" (P7).

Incorporate reflection on reasons to recover	1	"A thing that I think is a really critical part of treatment is motivational enhancement, and I think having list of pros and cons probably especially pros for changing, that would be available in the app, would be really really useful. I think a lot of what I do clinically is help people think about reasons for change and identify…where their eating disorder's been problematic and then…circle back to those again and again and again" (P10).
Include a toolbox of coping skills and lessons	2	"I feel like…having…games or a toolbox of coping skills could be helpful for increasing engagement" (P1).<br><br>"For example, if a patient is struggling with really high urges, having…a stress tolerance toolbox that are things that they could even do within the app itself that could help them engage in skills right then and there" (P1).<br><br>"So, I don't know how the psychoeducation will work on your app, but if it's…like, 'Here's a library of different topics that you can sort of read about at your leisure,' I would enjoy that" (P5).

Constantly update the app and incorporate feedback from users	2	"Just having a really well-developed, constantly updated app that takes feedback from both clinicians and the individuals using it. I think that would be a real deal breaker for tech, like I think with [tracking app] it works… and I can't give any feedback, and they're not making any changes, right, and that's frustrating because I think…there are clients who come up with some really good ideas about how we could change some things or ways that they wish they could make it more customizable or adaptable to suit their needs" (P9).
Increase the practicality of the app (ability to integrate assessment data, upload app data, etc.)	3	"I mean, either the app would have to do something practical, like email me a food diary…in such a way that…increases compliance. It could remind people to fill it out or prevent them from forgetting to bring their paper version. So, I would …use an app if it were practical, or I'd have to feel like it really enhances the treatment in some sort of meaningful way" (P5).<br><br>"From a provider side, I think that you want to have it provide you information in a relatively painless way that feels reasonably secure and that doesn't take a lot of effort on your part to get it going… and…in a perfect world allows you to upload some of the information to the app" (P10).<br><br>"If there was a way to integrate…assessment data in an app, I think that would be really helpful to be able to…track your patients' progress more overtime" (P6).
Very low technological burden; train users	2	"I'm always happy to have a training around 'OK here's our app; here's how to use it; here's a walkthrough. I'm here let's all download it together and I'll show you this feature, this feature, this feature,' because…there's a lot of people who might be hands on or visual learners, or…if we're wanting our clients to use it we should have an understanding of how to use it too" (P8).<br><br>"If you understand the app, then you can understand the clientele who will want to use the app and which clients you would want to refer [the app] to, instead of just blindly referring" (P8).<br><br>"As a provider, I would want the tech burden to be very, very low" (P10).

2 Appendix A

2.1 Interview Guide

The following guide reflects the types of questions that will likely be asked of participants during the interview. Some items may change due to the iterative nature of qualitative research. However, the overall topic and level of sensitivity will remain consistent.

1. What is available to your patients with anorexia nervosa when transitioning out of higher levels of care (this includes residential, inpatient, partial hospitalization, or intensive outpatient care).
    a. Do you provide a discharge treatment plan for your patients?

i. If yes: what does that plan look like? Is it required or recommended? b. What does post-acute care typically end up looking like for your patients? What's working? What can be improved?

c. Is it difficult to find quality outpatient providers for your patients? What barriers exist? (expensive, no trained providers, waitlists, difficulties connecting those from outside your area to care, etc.)

d. Are there ongoing supports or services that your center offers, beyond traditional treatment services? For example, do you utilize web or app-based resources or support groups?

e. Do all the patients with AN that are discharged from your treatment center follow through with outpatient therapy?

   i. If no: what percentage do not follow through?

f. What type of treatment do the outpatient therapists that your patients see upon discharge typically provide?

g. Where do your patients tend to be weight-wise when they discharge? Do they leave treatment with a specific weight goal or weight range?

2. Do you (or your center) have experience with the integration of digital technology into treatment? This could include integration of websites or apps into treatment, use of online guided self-help, or use of teletherapy.
   a. If yes: what specific technologies did you utilize?
   b. [For each digital technology used:]
   c. Can you tell me about your experience integrating [name of digital tech.]? What went well? What was difficult?
   d. Was it considered more of an add-on to treatment or a treatment in its own right?
   e. Who was responsible for the financial cost? (Patient or the center)
   f. Were any of these technologies specifically used to support patients in the post acute treatment period?
      i. If no: can you expand a bit on why not?
3. I noticed on the brief questionnaire you filled out ahead of our meeting that you indicated that your interest in incorporating specifically app-based interventions to support your patients with anorexia nervosa after they are discharged from higher levels of care was a ___ on our scale of 1 (very disinterested) to 5 (very interested).
   a. Can you tell me about why you chose that number?
   b. If less than 5: what would be helpful or necessary to increase your interest in integrating app-based interventions into treatment?
4. And then I also noticed that you indicated your comfort level regarding integrating app based interventions into post-acute treatment for anorexia nervosa was a ____ on our scale of 1 (very uncomfortable) to 5 (very comfortable).
   a. Can you tell me about why you chose that number?
   b. If less than 5: what would be helpful or necessary to increase your comfort with integrating app-based interventions into treatment?
5. I now want to transition and actually ask for your suggestions about an app that my team is currently developing to support individuals with anorexia nervosa in the post-acute period. The app is based on our existing app for BN and BED-type eating disorders and is being refined based on an established, in-person cognitive-behavioral therapy protocol for supporting individuals with anorexia nervosa after discharge from intensive treatment.

It is a guided self-help app, meaning that the user will have support from a coach. This will come in the form of asynchronous text-based communication as well as supplementary brief video chats (15-20 minutes each). The role of the coach is to provide motivation for the user to continue with the program, provide feedback on progress, and help with applying skills learned in the program to daily life. I will share my screen and show you a few screenshots of the app in its current form and offer a brief explanation of what our app has to offer, including a supplementary social networking feature. I will also show you a brief outline of the content we plan to cover in the app.

a. From this very brief review, what do you like about this app? What needs to be improved or changed in order for it to work for your population or at your treatment center?
b. With the knowledge that this is a coached app:
    i. Would your center have interest in providing the coaching?
    ii. What are your thoughts on social networking component of this program? iii. When do you think such an app should be introduced into your care flow? (e.g., toward the end of acute care, after discharge, etc.)
    iv. What kind of training do you think your center would need to integrate the app into your care flow?
c. Do you have any other suggestions for the app itself?
d. Do you have any other suggestions on how you could actually see this app being implemented in the real world/within your setting?

6. Is there anything you would like to share that we have not already discussed regarding the app or post-acute care?
